# Management of placenta accreta spectrum: Number 9 - September 2021

**DOI:** 10.1055/s-0041-1736371

**Published:** 2021-10-20

**Authors:** Álvaro Luiz Lage Alves, Lucas Barbosa da Silva, Fabrício da Silva Costa, Guilherme de Castro Rezende

**Affiliations:** 1 Faculdade de Ciências Médicas de Minas Gerais, Belo Horizonte, MG, Brazil; 2 Hospital Sofia Feldman, Belo Horizonte, MG, Brazil; 3 Maternal Fetal Medicine Unit, Gold Coast University Hospital and School of Medicine, Griffith University, Gold Coast, Queensland, Australia; 4 Faculdade de Saúde e Ecologia Humana, Belo Horizonte, MG, Brazil

## Key points

- Reducing caesarean section rates is the main preventive measure for the placenta accreta spectrum (PAS).
- Early diagnosis of PAS, adequate planning of surgical intervention and the use of effective techniques in intraoperative hemorrhagic control offer greater possibility of preserving life, the uterus and fertility.
- The treatment of PAS should be defined by a preoperative plan and performed in a tertiary service by a multidisciplinary experienced team.
- Maternal morbidity and mortality are lower among pregnant women with PAS treated in specialized centers.
- Ultrasound is the method of choice for the diagnosis of PAS, and standardized descriptors in grayscale, color Doppler and, if available, 3D power Doppler should be used.
- The most common ultrasound findings of the PAS are disruption of the interface between the uterine serosa and bladder walls and multiple intraplacental lacunae.
- Placental non-removal should be routine in the surgical treatment of PAS.
- Knowledge of the anatomical details and arterial components of the S1 and S2 genital vascular regions are of paramount importance for the surgical management of PAS.

## Recommendations

- All pregnant women with previous uterine surgery and low implantation placenta should undergo ultrasound evaluation at between 18 and 24 weeks of pregnancy.
- Delivery of stable patients with PAS should occur between 34 + 0 and 35 + 6 weeks. Antenatal use of corticosteroids is recommended. Interruptions even earlier than that are appropriate only if there are obstetric indications.
- Informed consent for treatment of PSA must be obtained, with discussion of all possible complications.
- Surgical management of PSA should be performed by professionals with experience in advanced pelvic surgery and skill in dissection of the parametrium, retroperitoneum and pelvic floor, bladder reconstruction, ureter reimplantation and uterine compression suture techniques and uterine and pelvic devascularization.
- Surgical preparation of the patient with PAS should include two large-caliber venous accesses, a central venous access, invasive blood pressure monitoring, pneumatic compression stockings, reserve of blood products and intensive care beds for the parturient and the newborn. Preoperative cystoscopy is not routinely recommended, although ureteral stents may be beneficial in placenta percreta with vesical trigone and/or parametrial invasion.
- Starting with spinal or epidural anesthesia until fetal extraction and proceeding to general anesthesia is a good anesthetic strategy, as wide dissection of vascular neoformations prolongs the surgical time.
- Placenta accreta spectrum, in its prior increta and percreta varieties, can be treated by segmental uteroplacental excision followed by restoration of the uterine anatomy or hysterectomy.
- In the surgical treatment of PSA, hysterotomy and fetal extraction should be performed outside the invaded uterine area, usually in the uterine fundus. Vascular neoformations must be carefully and selectively ligated, and removal of the affected myometrium or hysterectomy must be performed with the placenta in situ. In the face of placental invasion of the bladder, partial cystectomy and/or reimplantation of ureters may be necessary.
- In case of a surprise diagnosis of PAS, if the ideal surgical conditions are not present, the surgical procedure should be restricted to hysterotomy and fetal extraction outside the invaded uterine area, followed by hysterorrhaphy with the placenta in situ and laparorrhaphy. Definitive surgical re-approach should be performed within one to two weeks.
- If surgical treatment of PAS is impossible or at high risk for uncontrollable bleeding, maintenance of the placenta in situ with counseling about the inherent risks is an acceptable approach.

## Background

Postpartum hemorrhage (PPH) is the leading cause of maternal death worldwide. 1 Among the various specific etiologies of PPH, the PSA stands out for its relationship with its contemporary higher incidence and maternal morbidity and mortality. 2 The first histopathological description of PAS dates from 1937. 3 The higher incidence is correlated with the higher rates of cesarean sections and other surgical procedures in the uterus. Undoubtedly, this is the PPH etiology that imposes the greatest surgical difficulty, especially when neighboring pelvic organs are involved. 2

Reducing caesarean rates is the main preventive measure for PSA. In order to minimize its incidence, some authors have also recommended a high transverse hysterotomy in the first cesarean section, performed above the uterine segment. 4 5

In recent decades, advances have been made both in preoperative diagnostic accuracy by imaging methods and in surgical techniques related to PAS. When available, advance diagnosis, adequate planning of surgical intervention and the use of effective techniques for intraoperative hemorrhagic control offer a greater possibility of preserving life, the uterus and fertility. 4

### What should PAS screening and diagnosis be like?

Recent studies have shown that about half of PAS cases remain undiagnosed before delivery. 6 The antenatal diagnosis of PAS allows a multidisciplinary approach in the care for these pregnant women, reducing their morbidity by 50% and providing less blood loss and need for intrapartum blood transfusion. 7 Given its easy access and relatively low cost, ultrasound is the method of choice for the antenatal diagnosis of PAS. Its sensitivity and specificity are above 90%, and accuracy depends on the examiner's training and experience level. 6 Placenta accreta spectrum ultrasound signs may be present in the first trimester, even before 11 weeks, and the most common are implantation of the gestational sac in the anterior and inferior segment of the uterus and placental development near, over or within the scar of a previous hysterotomy. 8

The PAS is highly likely in pregnant women with placenta previa (and low anterior implantation) after one or more cesarean sections. 7 All pregnant women with previous uterine surgery and low anterior implantation of the placenta should undergo a complete transabdominal and/or endovaginal ultrasound evaluation of the interface between the placenta and myometrium, preferably between 18 and 24 weeks, with bladder repletion between 200 and 300 mL. 9 During transabdominal evaluation, excessive compression of the probe under the maternal abdomen should be avoided. 6 The use of selective screening protocols optimizes maternal and neonatal diagnosis and outcomes. 10

**Figure 1.**
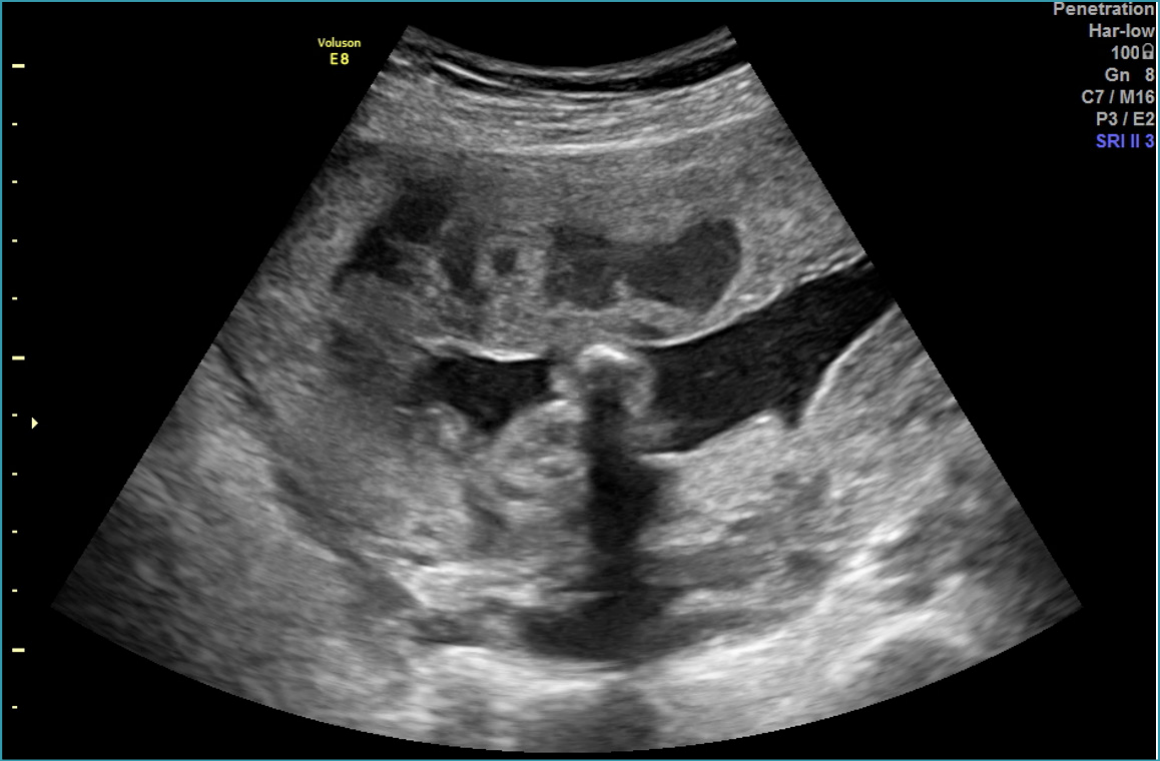
Grayscale ultrasound showing intraplacental hypoechoic images in the lower and anterior uterine segment compatible with placental lacunae in placenta previa accrete

**Figure 2.**
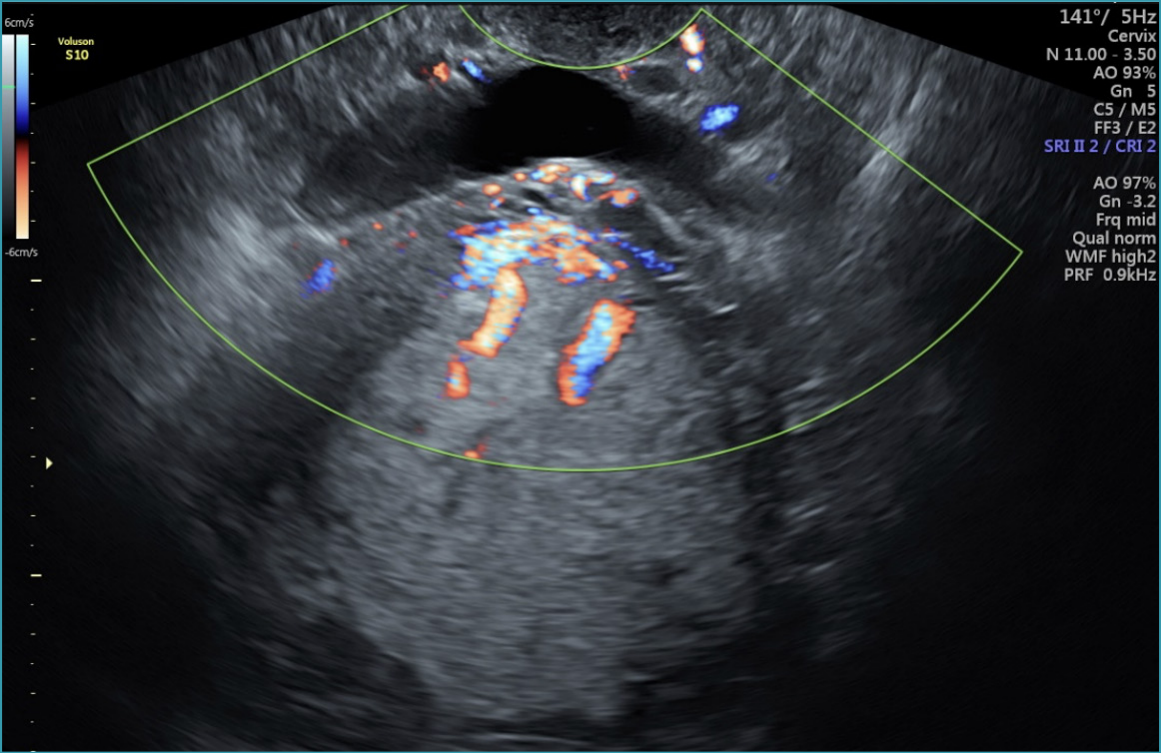
Cross-section of the uterovesical surface performed transvaginally with B-mode associated with color Doppler showing bridging vessels between placenta and bladder

**Figure 3.**
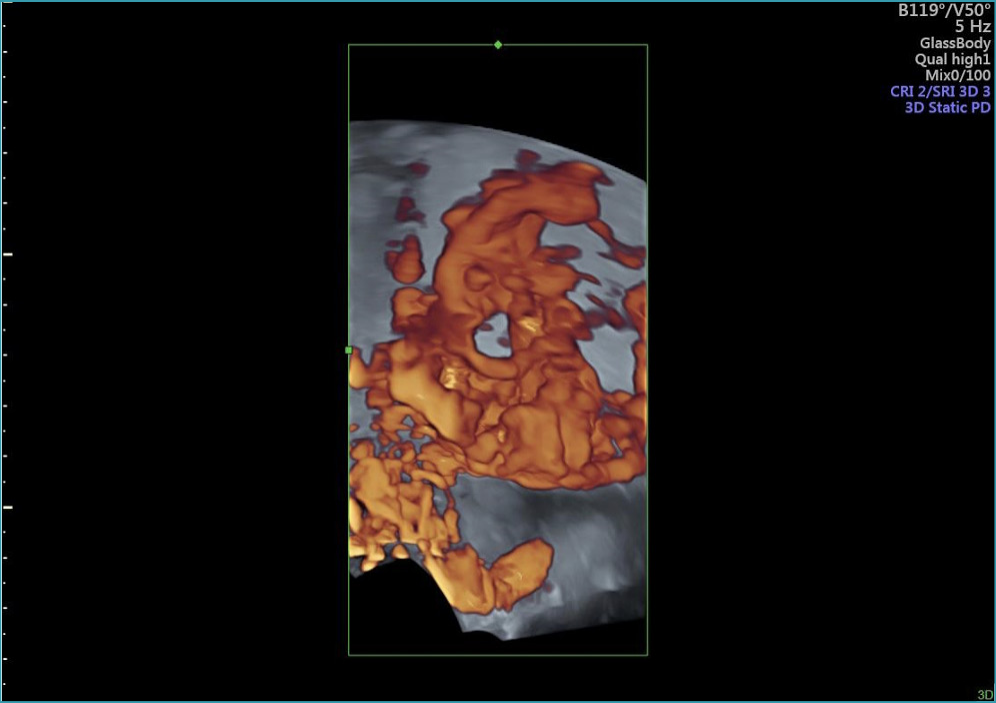
Three-dimensional rendered view of intraplacental hypervascularity associated with power Doppler

**Figure 4.**
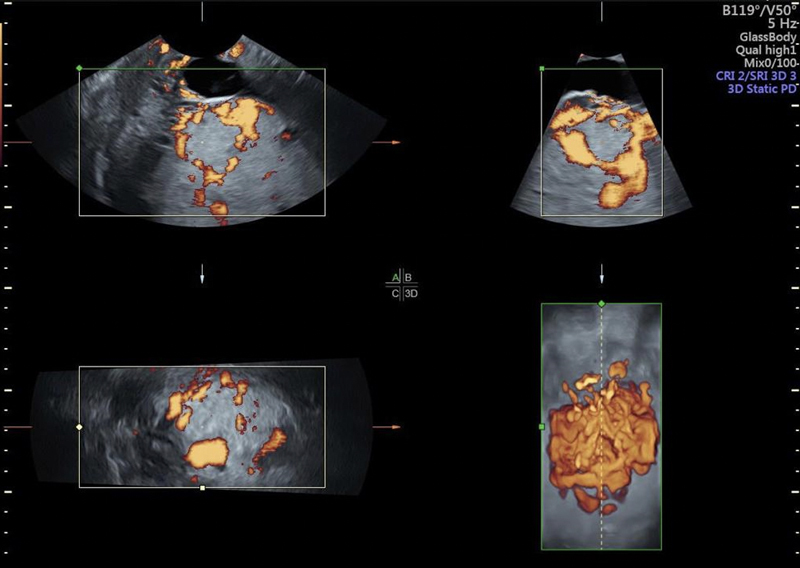
Multiplanar 3D representation of the placenta and uterovesical interface associated with power Doppler, showing uterovesical and intraplacental hypervascularity

Different ultrasound techniques have been used in the diagnosis of PAS in the second and third trimesters, especially grayscale, color Doppler and 3D power Doppler ultrasound. In order to reduce the subjectivity of the ultrasound diagnosis, the standardized findings are:

- Grayscale ultrasound;
  - Loss of the “hypoechoic area” under the placental bed;
  - Irregularity and attenuation of the uterovesical interface;
  - Reduced retroplacental myometrial thickness (<1 mm);
  - Placental bulge;
  - Exophytic masses reaching the uterine serosa;
  - Placental lacunae ( Figure 1 );
- Color Doppler:
  - Uterovesical hypervascularization (diffuse or focal intraparenchymal flows);
  - Subplacental hypervascularization (prominent venous complex);
  - Communicating vessels between placenta and bladder (bridging vessels) ( Figure 2 );
  - Communicating vessels with placental lacunae (high-velocity vessels with turbulent lacunar flow);
- Power Doppler 3D:
  - Intraplacental hypervascularity on 3D power Doppler ( Figures 3 and 4 ). 6

These terminologies and standardized criteria, aimed at distinguishing the different degrees of placental invasion, need to be more used in clinical practice. 11

Bridging vessels extend through the myometrium to the vesical serosa or other organs and should be distinguished from vesical varices commonly seen in normal pregnancies. It is essential to define if the placental invasion area is above or below the bladder trigone, and in the central region or on the lateral (parametrial) edge of the bladder, as lower and lateral invasions require the performance of complex surgical procedures in areas of difficult surgical access and hemostatic control, thereby increasing the risk of ureteral injury. 12 13

The role of other imaging methods in the diagnosis of PAS is uncertain. Magnetic nuclear resonance performed without gadolinium and preferably between 24 and 30 weeks is useful to detail the evaluations of the posterior placenta, the depths of the parametrial, myometrial and bladder invasions, and of the myometrium and placental portions laterally adjacent to previous hysterotomy. 14 15 In order to optimize the diagnosis, the findings should be interpreted in conjunction with ultrasound findings and by physicians experienced in PAS. 16 Three-dimensional power Doppler ultrasound can also contribute to the diagnosis of PAS, providing better details of irregular intraplacental vascularization and of the interface between the uterine serosa and bladder wall. 17

### How should the surgical intervention planning in the PAS be?

The treatment of PAS must be defined in a preoperative plan and instituted by a multidisciplinary team. The risk of severe intraoperative hemorrhage arising from vascular neoformations and invasive chorionic villi requires the performance of surgical approach in tertiary services by an experienced team, especially in placenta percreta that invade neighboring organs. 18 Therefore, the ideal is the presence of anesthetists, obstetricians and surgeons with experience in oncogynecological surgery, neonatologists, hematologists and blood bank staff, interventional radiologist, intensivists and the respective specialized nursing teams. 19 20

Informed consent must be provided with discussion of all potential complications (blood transfusion, urinary and/or intestinal lesions, urinary and/or intestinal fistula, hysterectomy, etc). The surgeon must have experience in advanced pelvic surgery, knowledge in parametrial, retroperitoneal and pelvic floor dissection, bladder reconstruction, ureter reimplantation and uterine compression suture techniques and uterine and pelvic devascularization. 21

Surgical planning should include reserve of blood components, selection of the most experienced professionals available, review of the invaded genital vascular region and definition of anesthetic technique and laparotomic incision. Delivery of stable patients should be planned for the gestational age between 34 + 0 and 35 + 6 weeks. 2 This anticipation is justified, since the placental blood flow in the pregnancy term in the PAS is 600-700 mL/min. 20 The use of antenatal corticosteroids is recommended in accordance with standard guidelines. Even earlier interruptions are only appropriate in the case of obstetric indications (heavy vaginal bleeding, premature amniorrhexis, and high risk of preterm delivery). 2

Two large-caliber venous accesses should be provided, a central venous access, invasive blood pressure monitoring, pneumatic compression stockings, reserve of blood products (massive transfusion protocol) and intensive care beds for the mother and newborn. Preoperative cystoscopy is not routinely recommended, as it does not increase the accuracy of imaging tests in identifying bladder invasion, even in the presence of hematuria. However, ureteral stents (double-J) can be beneficial, especially in placenta percreta with lower invasion (bladder trigone, parametrium), reducing the risk of inadvertent injury to the ureters, whose parametrial and paracervical anatomy may be altered by placental invasion. 21

Endovascular radiological intervention through the insertion of a balloon catheter in the internal iliac arteries (hypogastric) and/or embolization of the uterine arteries and/or internal pudendal arteries can be used to reduce perioperative bleeding. 21 22

### Is the pelvis anatomy altered in the PAS, exerting influence on the surgical approach?

**Figure 5.**
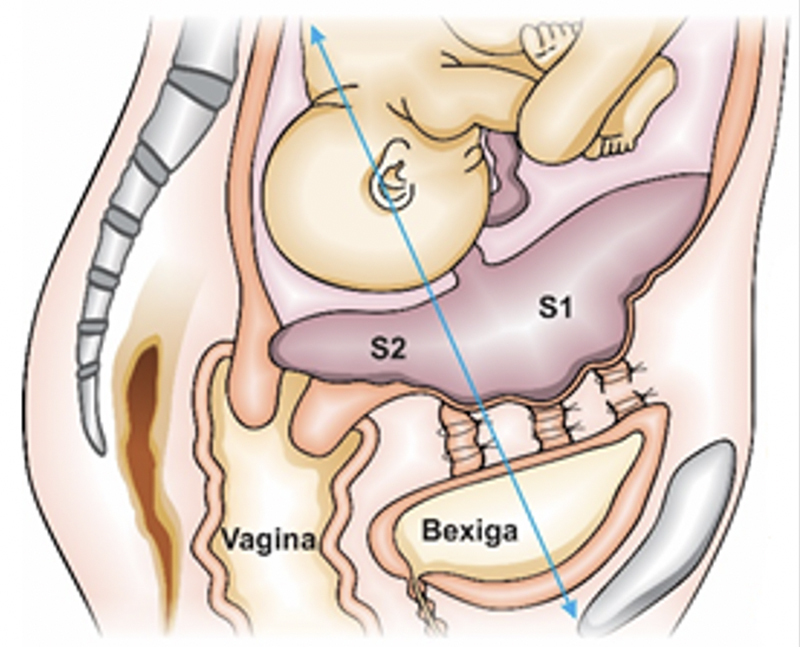
Sagittal diagram of the division of S1 and S2 genital vascular regions. Source: Illustration by Felipe Lage Starling (authorized), 2021.

Knowledge of the anatomical details of the arterial components that irrigate the uterus and its adnexa, as well as their anatomical and anastomotic varieties is of paramount importance for the surgical approach to PAS. In a sagittal section of the female pelvis, a perpendicular imaginary line drawn at the level of the middle sector of the posterior bladder wall identifies two distinct vascular areas in the reproductive system. The upper area, called the S1 genital vascular region, includes the uterine fundus and body. This region is irrigated by uterine and ovarian arteries, which favors the success of uterine devascularization techniques and uterine compression sutures. The lower area, called the S2 genital vascular region, is formed by the lower uterine segment, cervix and upper part of the vagina. In PAS, this region receives blood supply from the internal pudendal, inferior vesical, and middle, superior and inferior vaginal arteries, and an anastomotic system is present between the vaginal and uterine arteries ( Figure 5 ). This explains the ineffectiveness of traditional hemostatic mechanisms in the S2 region and the need for specific procedures for hemorrhagic control. 19

**Figure 6.**
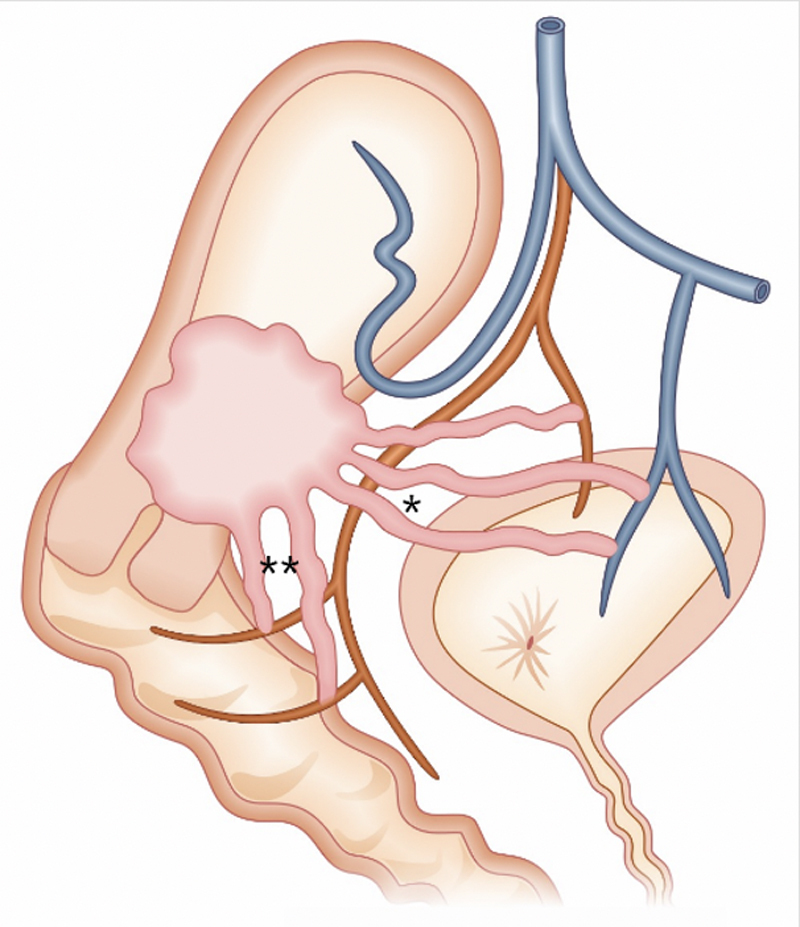
Vesicouterine, vesicoplacental and colpouterine anastomotic systems. Source: Illustration by Felipe Lage Starling (authorized), 2021. *Vesicouterine and vesicoplacental anastomotic systems. ** Colpouterine anastomotic system

The disordered neovascularization present in the PAS is composed of placental vessels with absent or rudimentary tunica media (muscular), limiting the success of hemostasis by electrocoagulation. In addition, particles used in embolization procedures can cross vascular walls and necrotize extrauterine tissues. The subperitoneal vascular pedicles (vaginal, inferior vesical and internal pudendal arteries) that irrigate the S2 region are difficult to surgically access and lead to occult retroperitoneal hemorrhage. In addition, there are three anastomotic systems communicating the vasculature of the uterus, placenta and adjacent organs. The vesicouterine system has anastomoses between the uterine arteries and the super-posterior portion of the bladder (vesicouterine fold). The vesicoplacental system has anastomoses between the placenta and the detrusor muscle, which can be identified on ultrasound. The colpouterine system is less known and presents the greatest difficulty for surgical access, interconnects the retrovesical space to the anterior vaginal wall, parametrium and paracolpos, promoting the formation of varicose vessels along the vaginal axis, deep pelvis and pelvic floor ( Figure 6 ). Thus, hemostatic control of these areas is more safely achieved through double ligations, as embolization can provide hemorrhagic control of the vesicouterine and vesicoplacental systems, but not of the colpouterine system. Segment compression sutures are an alternative for hemorrhagic control in this area. 19 20

### How should be the surgical management of PAS diagnosed in advance?

Anesthesia can be general, blockade or combined. Starting with spinal anesthesia or epidural anesthesia until fetal extraction and sequentially proceeding to general anesthesia is a good strategy, as extensive dissection of vascular neoformations inevitably prolongs the surgical time. 19 Continuous epidural analgesia with appropriate preparation for conversion to general anesthesia is also a good option. 23

The patient should be placed in a lithotomy position, the uterus shifted to the left and legs apart to allow vaginal access during surgery. Laparotomy should be wide (Cherney, Maylard) and longitudinal incisions may be necessary for adequate surgical exposure. After extensive uterine exposure, hysterotomy and fetal extraction should be performed outside the invaded uterine area. Therefore, uterine fundal hysterotomy should be performed, which can be done in the anteroposterior (Caruso) or transverse (Fritsh) directions. Perioperative ultrasonography, with the probe protected by a sterile glove may help to identify the placental border, better defining the site of incision. After fetal extraction and clamping with removal of the umbilical cord, hysterorrhaphy is performed and the placenta should be kept in situ. The ureters and internal iliac arteries must be located and the surgical technique defined. 19

**Figure 7.**
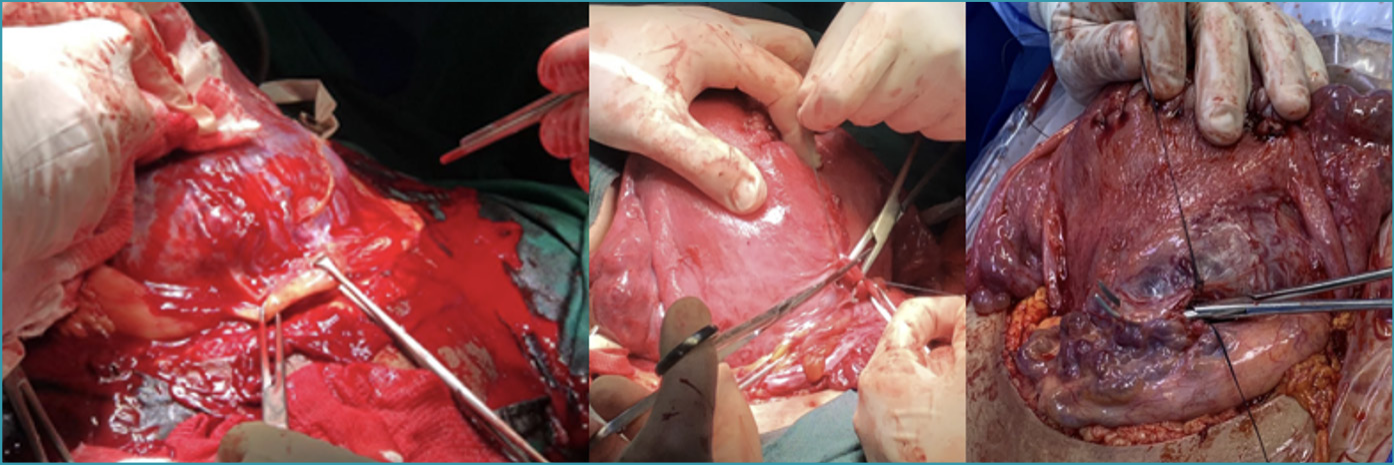
Low selective ligations of vascular neoformations present in the uterine segment in the surgical management of placenta accreta. Exposure of vascular neoformations present in the vesicouterine reflection by means of traction with Allis forceps. Double ligations made using a suture passer

**Figure 8.**
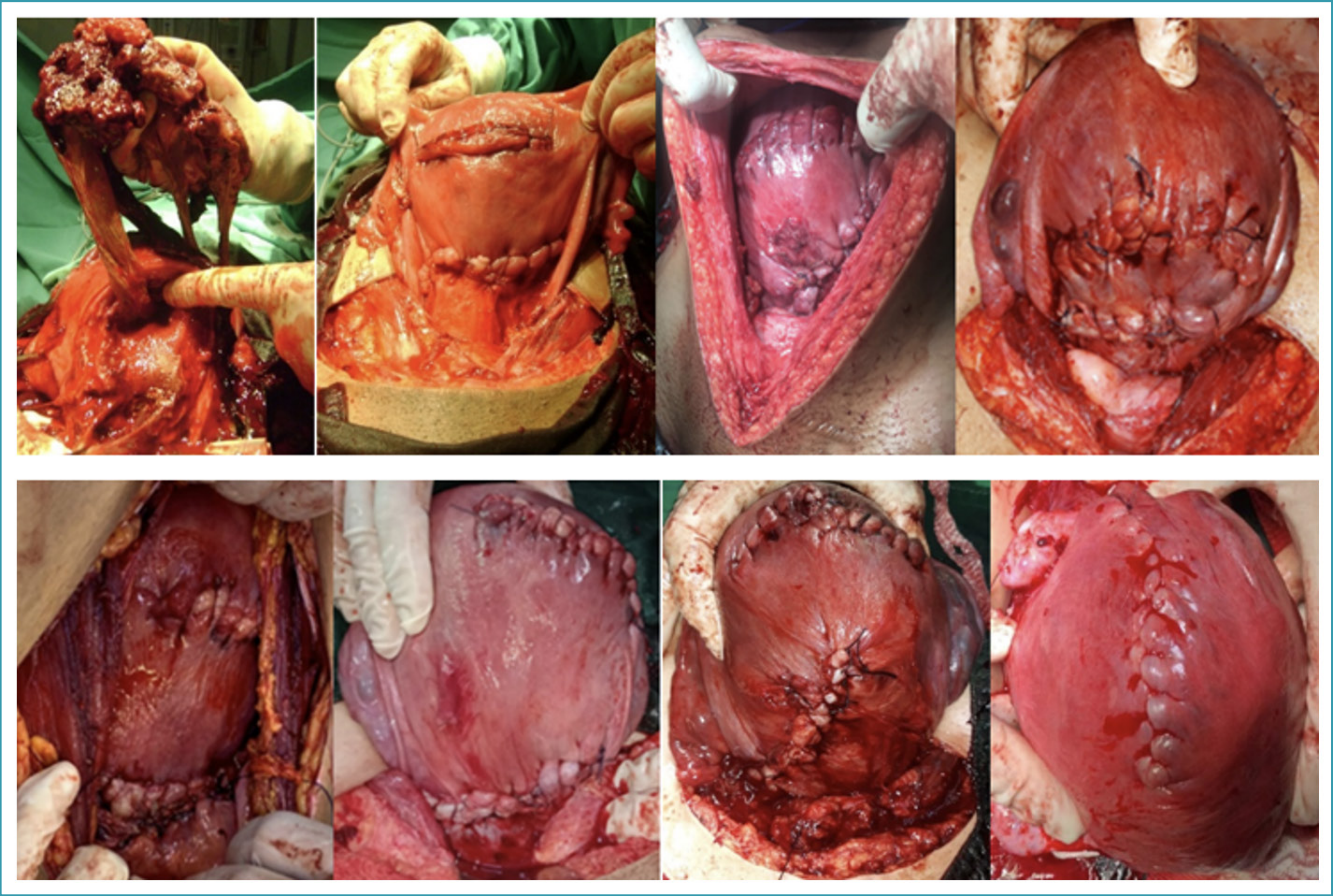
Excision with uteroplacental segmental exeresis followed by restoration of the uterine anatomy in conservative surgical treatment of placenta accreta. Upper left – excision of the uterine segment affected by invasion of placental cotyledons and ovular membranes. Other images – final aspects of the restoration of the uterine anatomy with hysterorrhaphy in the fundus or uterine body and suture between the uterine body and the residual lower uterine segment

Excision with uteroplacental segmental excision followed by restoration of the uterine anatomy (conservative surgery), should be preferred to hysterectomy. In addition to potentially preserving fertility, it has the advantage of minimizing intraoperative hemorrhagic loss. This technique provides the surgical disconnection of the invaded organs (uterus, placenta and bladder) through dissection and appropriate exposure of the pelvic compartment and its avascular spaces, and execution of hemostatic ligatures, complete resection of the invaded myometrium and uterine and/or bladder reconstruction. Both conservative surgery and hysterectomy require surgeon experience and skill to perform low selective ligatures of vascular neoformations present in the uterine segment (vesicouterine, vesicoplacental and colpouterine anastomotic systems). The use of suture passer facilitates the careful execution of double ligations, obstructing the blood flow in the vascular neoformations present ( Figure 7 ). Bladder dissection must be thorough, with ligation and section of all the communicating vascularization between its posterior wall and the uteroplacental interface until reaching the upper third of the vagina. 19 This dissection can be facilitated when performed bilaterally through paravesical spaces, preferably with half-full bladder (200 to 300 mL). 24 Usually, the bladder area with the greatest placental invasion is the central and apical portion, which is less vascularized. Since invasions of the lateral parametrium and bladder trigone are rare, dissection of the vesicouterine peritoneum and bladder detachment from the uterine wall through its lateral border, through the paravesical spaces, are easier and safer. The dissection must proceed laterally and inferiorly, until visualization of the vesical insertion of the ureters and the superior vaginal portion. This dissection, performed in a blunt and delicate way, provides a “tunnel” communicating the right and left lateral borders of the uterus, communicating the fingers of both hands, posteriorly to the bladder (“ *Pelosi bypass* ”). 25 After finishing the ligation of vascular neoformations, the entire uterine segment invaded by placental cotyledons is excised without previous attempt to remove them from the myometrium. The restoration of the uterine anatomy is achieved through a suture applied between the lower part of the uterine body and the lower residual portion of the segment ( Figure 8 ). 19

Since the scar tissue is removed together with the placental cotyledons, ovular membranes and affected myometrium, the incidence of PAS in subsequent pregnancies is not significant. 19 Even so, these patients should be advised about the subsequent risks (PSA, uterine rupture) and undergo early screening for placenta accreta in subsequent pregnancies. 26

**Figure 9.**
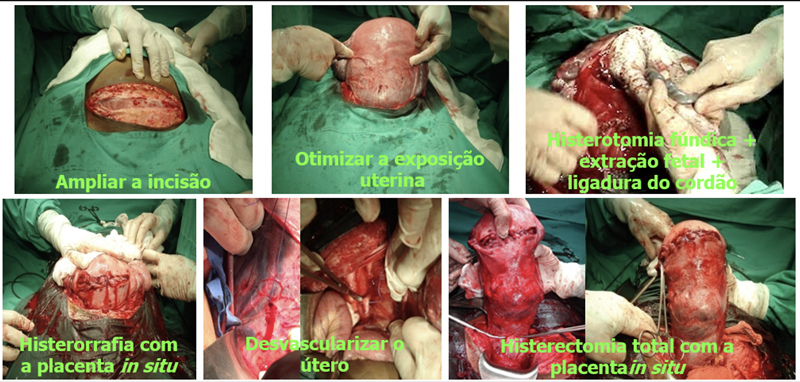
Steps of the cesarean-hysterectomy technique in the surgical management of placenta accreta

**Figure 10.**
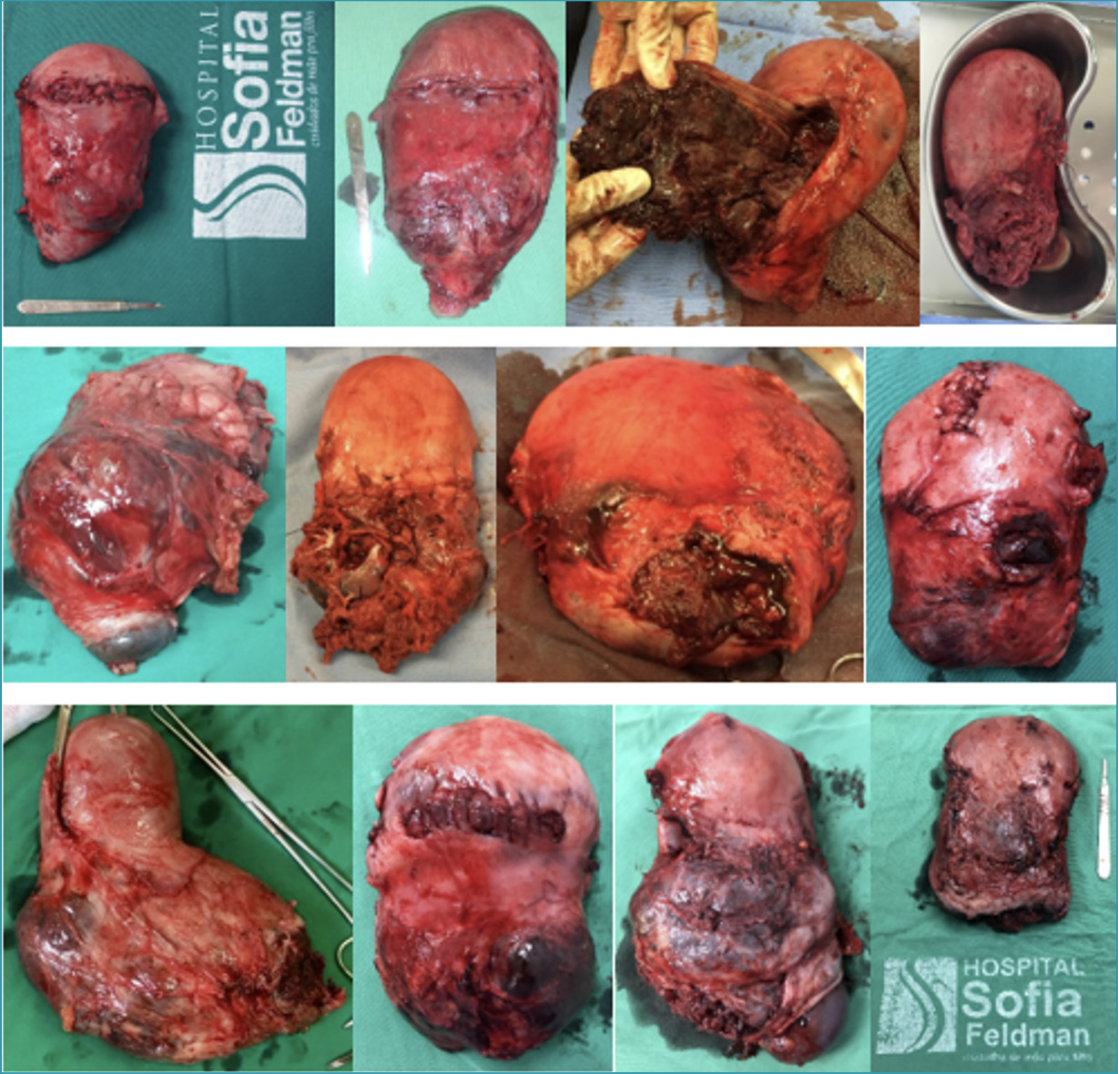
Non-conservative surgical treatment of placenta accreta. Final aspects of uteri removed with placentas in situ in cesarean-hysterectomy

**Figure 11.**
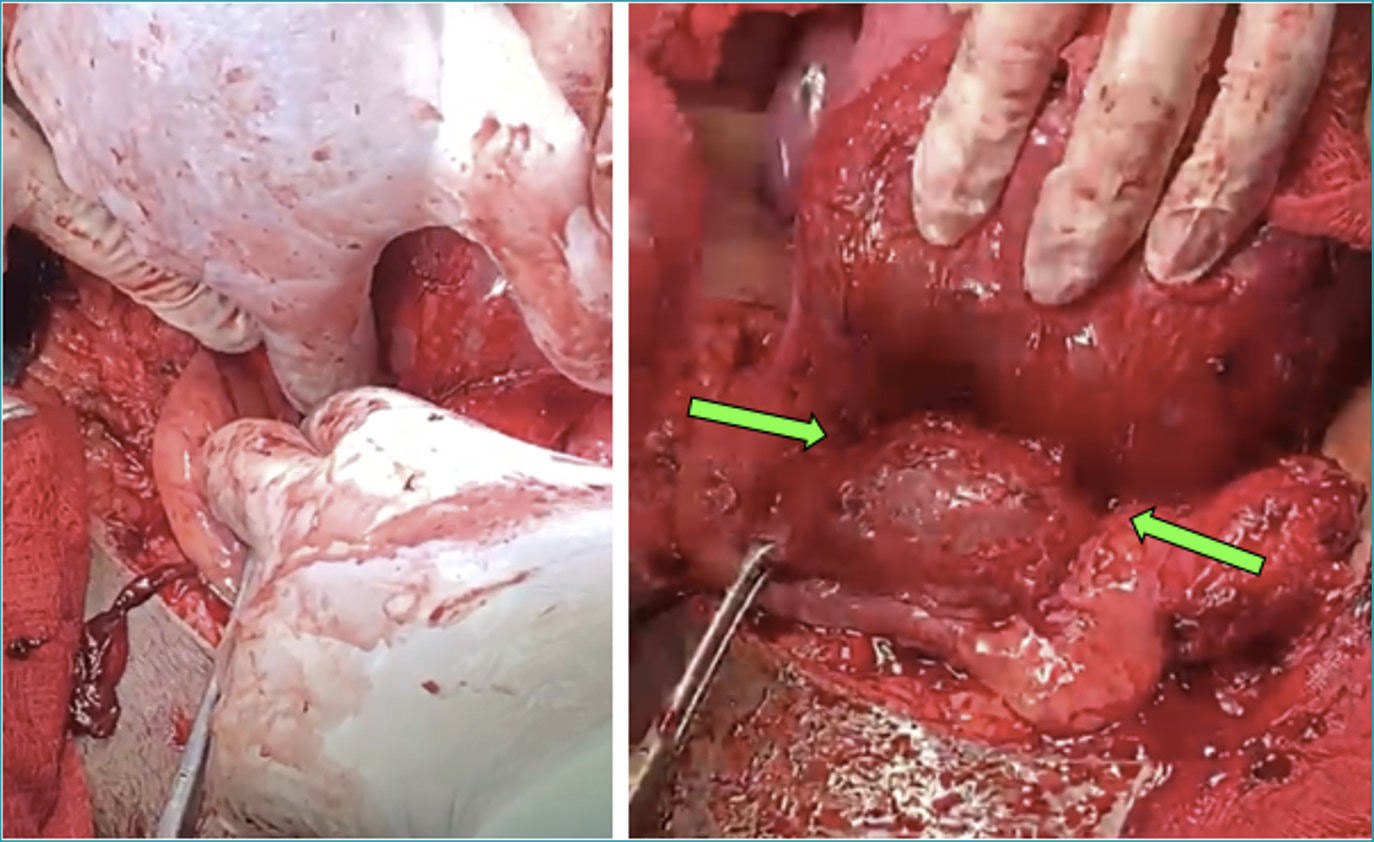
Bladder mobilization and dissection (Pelosi bypass) performed in the areas of vesicouterine adhesions in the surgical treatment of placenta accreta. Green arrows - after performing low selective ligations of vascular neoformations, mobilization and blunt dissection of the vesicouterine space are performed

Although hysterectomy is a definitive treatment, it imposes an additional blood loss of 2 to 3 liters, providing mean surgical loss between 3 and 5 liters and the need for blood transfusion in 90% of patients. Parametrial and cervical invasions are indicative of total hysterectomy. Invasions above 50% of the axial uterine circumference and segmental tissue loss with a permanence of less than 2 cm of healthy tissue above the cervix are also indicative of uterine removal, as they make it impossible to adequately reconstruct the anterior uterine segment, with a high probability of ischemia, infection and necrosis. 21 The uterus must be removed with the placenta in situ. If placental invasion of the isthmo-cervical region is in force, hysterectomy must be total, as maintenance of the uterine cervix is associated with postoperative hemorrhagic recurrence. The uterus is devascularized immediately after hysterorrhaphy, before ligation repairs. Ligations of the ascending branches of the uterine arteries are performed in the utero-ovarian connections of the mesosalpinx, in the cervicouterine arteries and in the vascular neoformations present in the uterine segment, controlling hemorrhage in the S1 and S2 genital vascular regions. Optionally, bilateral ligation of the internal iliac arteries can be included in the devascularization technique ( Figures 9 and 10 ). In hysterectomy performed with high vascularization and uterovesical adhesion, mobilization and bladder dissection (“Pelosi bypass”) performed in areas of adhesions are useful in preventing urinary tract injuries ( Figure 11 ). 19 25

**Figure 12.**
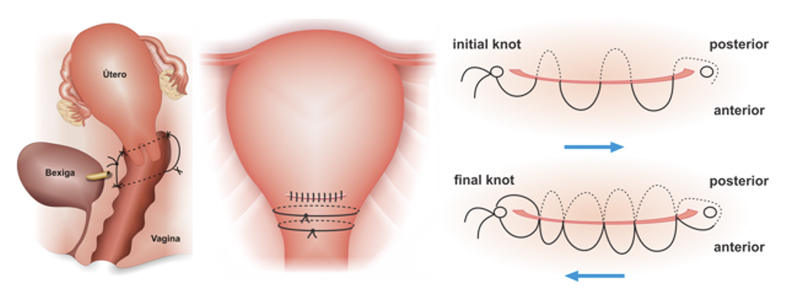
Uterine compression sutures of Cho (adapted by Palacios-Jaraquemada), 20 Dedes and Ziogas 27 and segment transverse sutures in multiples of eight. Source: Illustration by Felipe Lage Starling (authorized), 2021.

In the presence of placental invasion of the vesical fundus, one option is to perform partial cystectomy and one-piece hysterectomy (Pelosi technique). 25 Eventually, ureteral reimplantation is necessary. An alternative aiming at hemorrhagic control in the S2 genital vascular region is the application of segment compression sutures. The most indicated techniques for this purpose are Cho (adapted in S2 by Palacios-Jaraquemada), 20 Dedes and Ziogas 27 or the segment transverse suture in multiples of eight ( Figure 12 ). 20 27 28 28

The strategies described above offer the advantage of one-step surgical resolution. 19 20 Intensive care should be provided in the postoperative period, as the continuation of hemotherapy, invasive hemodynamic monitoring, ventilatory support and the use of vasopressors are often necessary. In case of postoperative bleeding, interventional radiology can provide embolization of deep pelvic vessels, avoiding surgical re-approach. 30

### How should the surgical management of PAS be in case of a surprise diagnosis?

The absence of the PAS antenatal diagnosis is invariably linked to the lack of appropriate screening in pregnant women with risk factors. During cesarean section, the surprise diagnosis of PAS can be visual or by the difficulty in placental removal. In the more invasive placenta (increta and percreta), vascular neoformations and chorionic villi reaching the deep myometrium and reaching or exceeding the uterine serosa are identified without much difficulty. In accretism restricted to the basal decidua, suspicion often occurs because of the difficulty in removing the placenta, since changes in the uterine serosa are absent. In the vaginal delivery route, the diagnostic suspicion occurs if placenta is retained beyond 30 minutes of birth. 20

Upon the surprise diagnosis of the PAS, the first approach to be adopted is not to try to remove the placenta. Improper removal will invariably lead to massive hemorrhage and rapid onset of the lethal triad (coagulopathy, metabolic acidosis and hypothermia). Therefore, when PAS is suspected, the surgery should be interrupted briefly with the aim of providing blood components, re-discussing and reorganizing anesthetic and surgical procedures, and expanding the incision for adequate pelvic exposure. 19 20

The surgical technique should also be guided by the identification of the genital vascular regions (S1 and S2) affected by placental invasion. The chosen approach should be the most likely to avoid massive intraoperative hemorrhage. The surgical management options are the same, that is, hysterectomy or uteroplacental segmental excision, followed by restoration of the uterine anatomy (conservative surgery). In non-ideal surgical conditions (lack of experience of the team and/or blood components), the surgical act should be restricted to hysterotomy and fetal extraction outside the invaded uterine area, followed by hysterorrhaphy with the placenta in situ and laparorrhaphy. In these situations, the definitive re-approach (two step) will be performed within one to two weeks after the complete reorganization of care conditions. Despite the demerit of multiple surgical procedures, the reduction in intrauterine pressure provided by fetal extraction induces the collapse of the newly formed vessels and a slight edema in the vesicouterine reflection. These modifications facilitate tissue dissection and reduce the chances of bleeding during the definitive re-approach. 19 20

### When is placental removal without hysterectomy or conservative surgery acceptable in the treatment of PAS?

**Figure 13.**
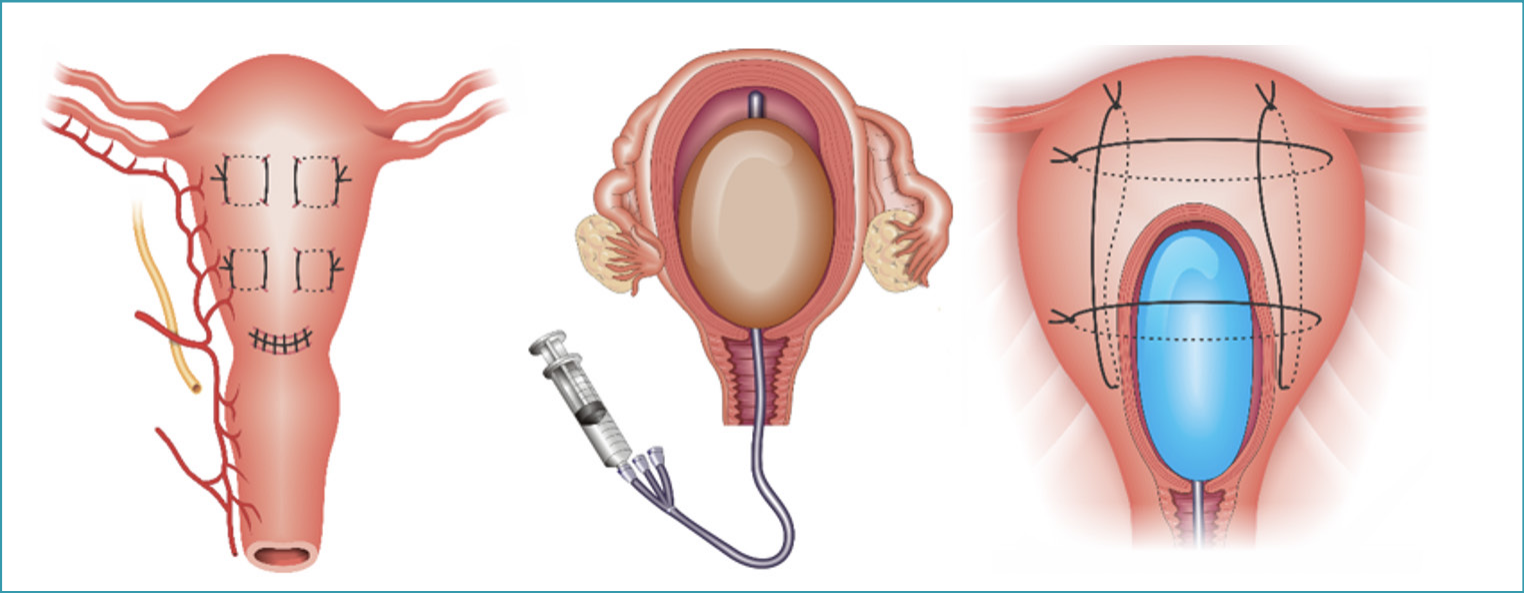
Cho's uterine compression suture, uterine balloon tamponade and uterine sandwich technique. Source: Illustration by Felipe Lage Starling (authorized), 2021.

The risk of uncontrollable hemorrhage and intraoperative evolution to the lethal triad is significant in view of the attempt to remove placenta in the PAS, especially in placenta previa implanted in the anterior uterine segment with a high degree of invasion and neovascularization. Therefore, the general guideline is not to remove placental during surgical management of PAS. 19 20 However, in selected cases of focal accretism and posterior or fundic placenta, placental removal with uterine preservation can be successfully performed without major risks. Patients with focal accretism with an area of adhesion less than 50% of the anterior surface of the uterus and with healthy and accessible myometrial borders are the best candidates for this approach. In posterior placenta or implanted in the uterine fundus, bleeding from placental removal is easier and faster to be controlled, which makes this approach also viable in these situations. 21 Cho's uterine compression suture applied in S1 or S2 is the most indicated surgical technique for uterine preservation for adjuvant use in these cases. In uteri without involvement (fragility) of the myometrial wall, uterine balloon tamponade may also be associated. Optionally, the balloon can be used in association with a uterine compression suture (uterine sandwich technique) ( Figure 13 ). 1 2

### When and how to institute non-surgical PAS treatment?

**Figure 14.**
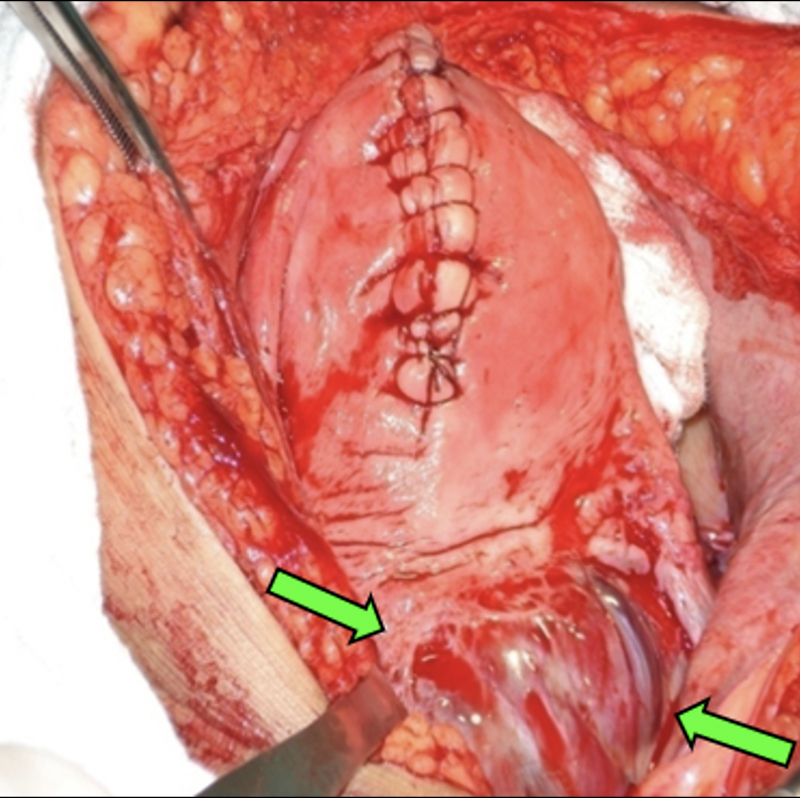
Placenta previa percreta implanted in the iliac vessels. Green arrows - placental tissue and vascular neoformations implanted over the right iliac vessels

In situations where the surgical management of PAS is considered high risk or impossible for uncontrollable hemorrhage, maintaining the placenta in situ is an acceptable and exceptional conduct, even if associated with several risks. Extensive invasions of the bladder (bladder trigone), cervix, parametrium or other neighboring organs and implantation of cotyledons in the great pelvic vessels are the most common clinical presentations that justify this approach ( Figure 14 ). In these situations, surgical intervention should be limited to hysterotomy outside the invaded area, extraction of the fetus, umbilical cord and ovular membranes, hysterorrhaphy with the placenta in situ and laparorrhaphy. 31 Uterotonics, uterine compression sutures, uterine compression with bandages, vascular ligations and arterial embolization may be associated with the procedure. Broad-spectrum antibiotic prophylaxis has been recommended, but without proven benefits. Methotrexate has no longer been recommended, as there is a lack of evidence regarding its efficacy and clear evidence of damage (pancytopenia, nephrotoxicity). 21 Hysteroscopy for late resection of the ovular material can be performed. 32 These patients should be monitored clinically for weeks to months, as the rate of placental absorption and expulsion is uncertain. They should also be extensively counseled about possible short-term complications: hemorrhagic recurrence, infection, last-resort hysterectomy, and death. 31 Intrauterine synechiae and secondary amenorrhea are possible late complications, but subsequent pregnancies are feasible in most patients and recurrence of PAS ranges between 22% and 29%. 21 26

### What are the other adjuvant procedures in the treatment of PAS?

Intraoperative cell salvage can be used to rapidly supply large amounts of autologous blood during surgical management of PAS, helping to reduce allogeneic blood transfusion. The procedure includes autotransfusion after leukocyte filtration, washing and centrifugation, requiring specialized technology and professionals. The risks (maternal infection, amniotic fluid embolism, alloimmunization) are minimal and lower than those of allogeneic transfusion or similar to these. 1 33

When available, endovascular radiological interventions can be used to reduce bleeding in the surgical field. Techniques include insertion of a balloon catheter into the internal iliac arteries, embolization of the uterine and/or internal pudendal arteries, or a combination of both. They are also alternatives to partial cystectomy when there is bladder invasion, especially of the trigone. Since there is evidence of reduction of intraoperative bleeding, but not of the need for blood transfusion, the consideration of these procedures remains controversial and it is still impossible to predict which patients will benefit from these techniques. 21 In embolization, Gelfoam® particles are administered after fetal extraction with the intention of temporarily occluding the flow in the uterine arteries and/or internal pudendal arteries. In balloon occlusion, catheters are inserted into the internal iliac arteries preoperatively under fluoroscopic guidance. After fetal extraction, balloons are inflated intermittently for 20 minutes. Balloon catheters have the advantage of being kept for several hours in the postoperative period, and can be inflated again in case of hemorrhagic recurrence. 22 Temporary balloon catheter occlusion of the common iliac arteries has also shown good results in situations of severe hemorrhage. The technique blocks the anastomotic component of the femoral artery to the pelvis, as well as the internal pudendal artery, as it interrupts the blood flow in the posterior division of the internal iliac arteries. 34

In the face of predictability or presence of massive hemorrhage, cross-clamping of the infrarenal aorta reduces uteroplacental blood flow and intraoperative blood loss, technically facilitating the ligation of vascular neoformations. The procedure must be performed after fetal extraction and hysterorrhaphy. Move the uterus inferiorly to improve exposure. The retroperitoneum must be opened between the inferior mesenteric artery and the bifurcation of the aorta. The areolar tissue surrounding the aorta must be dissected and the aorta must be separated from the inferior vena cava. With the aid of a suture passer, a bandage is passed under the aorta, surrounding it, in order to elevate the vessel and facilitate the application of the clamp. A flexible, atraumatic cardiovascular clamp is the most suitable device for clamping, and should be applied with minimal fixation force. Ideally, the duration of clamping should be less than 60 minutes and a pulse oximeter should be installed to monitor arterial O _2_ saturation throughout the procedure. This technique must be performed by a well-trained surgeon. 35 A more rational strategy for proximal vascular control is temporary manual occlusion of the infrarenal aorta, a simple and quick procedure. After exteriorization of the uterus and displacement of the sigmoid colon to the left, the aortic bifurcation is visualized over the promontory. A simple manual pressure of the aorta against the spine immediately stops the blood flow. 36

Ligation of the internal iliac arteries has limited effectiveness (40%), as immediately after occlusion, a network of collateral circulations is established, involving the lumbar, ileolumbar, middle and lateral sacral, and middle and superior rectal arteries. However, it can be useful as an adjunct to pelvic packing in situations where damage control is instituted. 37

Another adjunct method indicated for patients undergoing conservative surgery with established coagulopathy is the application of an external elastic bandage to the uterus. After application of uterine compression suture and fibrin sealant, the uterus is wrapped with one or two elastic bandages (Esmarch®), applied from the fundus to the cervix, and the patient is subjected to damage control (pelvic packing and laparostomy). 38

### What are the necessary criteria to make a center of excellence in PAS viable?

Maternal morbidity is demonstrably lower among pregnant women with PAS treated in specialized centers with proven experience. 39 Despite the lack of consensus on the definition of a center of excellence in PAS and the minimum number of patients to be treated annually, the main suggested criteria are:

1. Multidisciplinary team:
  - Maternal-fetal medicine specialist obstetrician;
  - Specialists (ultrasound specialists, radiologists) in imaging exams (Doppler and three-dimensional ultrasound, nuclear magnetic resonance);
  - Pelvic surgeon (gynecological oncology or urogynecology);
  - Anesthesiologist;
  - Urologist;
  - General or trauma surgeon;
  - Interventional radiologist;
  - Neonatologist;
2. Intensive care unit (ICU) and facilities:
  - Interventional radiology;
  - Adult ICU;
  - Neonatal ICU;
3. Blood bank:
  - Capacity for massive transfusion;
  - Cell salvage and perfusionists;
  - Experience and access to alternative blood products;
  - Guidance from specialists in transfusion medicine or hematologists. 2 21 30 40

## Final considerations

In recent decades, childbirth care has evolved with new contradictions in its paradigms, based on the decline in the acquisition and use of childbirth care skills and greater safety of cesarean sections, culminating in a dizzying increase in the rates of this procedure and the insurrection of PAS epidemics in several world territories. Consequently, the increasing incidence and marked lethality associated with PAS challenge the actions to reduce maternal mortality from postpartum hemorrhage more than any other etiology. The screening, diagnosis and treatment of PAS require access to tertiary services, multidisciplinary care and acquisition of knowledge and skills, which are mostly “soft-hard technologies” that emerged and evolved because of the need to reduce the severe morbidity and preserve the fertility of affected patients. Therefore, it has become essential that obstetricians, pelvic surgeons, ultrasonographers, anesthesiologists and radiologists become familiar with the new procedures that should be incorporated in the care of these patients, such as three-dimensional ultrasound, nuclear magnetic resonance, intravascular balloons, pelvic vessel embolization, uterine compression sutures, vascular ligations, uterine bandaging, damage control techniques, among others. It is also necessary that surgeons become skilled in surgical tactics capable of minimizing the intraoperative bleeding present in hysterectomies and uteroplacental segmental excisions, as well as in performing partial cystectomies and ureteral reimplantations. Finally, appreciating women's lives and the planning and reorganization of care flows with the organization of reference centers for PAS care, implementation of risk stratification, and availability and training of teams for the correct use of these new technologies are the milestones of this most recent care challenge when facing postpartum hemorrhage.

National Specialty Commission in Obstetric Emergency of the Brazilian Federation of Gynecology and Obstetrics Associations (FEBRASGO)

President:

Alvaro Luiz Lage Alves

Vice-President:

Gabriel Costa Osanan

Secretary:

Samira El Maerrawi Tebecherane Haddad

Members:

Adriana Amorim Francisco

Alexandre Massao Nozaki

Brena Carvalho Pinto de Melo

Breno José Acauan Filho

Carla Betina Andreucci Polido

Eduardo Cordioli

Frederico Jose Amedee Peret

Gilberto Nagahama

Laises Braga Vieira

Lucas Barbosa da Silva

Marcelo Guimarãs Rodrigues

Rodrigo Dias Nunes

Roxana Knobel

National Specialty Commission on Ultrasonography in Gynecology and Obstetrics of the Brazilian Federation of Gynecology and Obstetrics Associations (FEBRASGO)

President:

Eduardo Becker Júnior

Vice-President:

Heron Werner Júnior

Secretary:

Sergio Kobayashi

Members:

Adriana Gualda Garrido

Anselmo Verlangieri Carmo

Fabrício da Silva Costa

Fernando Maia Peixoto Filho

Guilherme de Castro Rezende

Joffre Amim Junior

Jorge Roberto Di Tommaso Leão

Luciano Marcondes Machado Nardozza

Luiz Eduardo Machado

Manoel Alfredo Curvelo Sarno

Patricia El Beitune

Pedro Pires Ferreira Neto
